# Thymic stromal lymphopoietin (TSLP) acts as a potent mucosal adjuvant for HIV-1 gp140 vaccination in mice

**DOI:** 10.1002/eji.201141787

**Published:** 2011-11-07

**Authors:** Griet A Van Roey, Mauricio A Arias, John S Tregoning, George Rowe, Robin J Shattock

**Affiliations:** 1 Centre for Infection and Immunity, Division of Clinical Science, St. George's University of LondonCranmer Terrace, London, UK; 2 Mucosal Infection & Immunity Group, Section of Infectious Diseases, Imperial College LondonSt. Mary's Campus, London, UK

**Keywords:** Cytokine, HIV, Intranasal, Mucosal vaccine

## Abstract

The development of a successful vaccine against HIV is likely to require the induction of strong and long-lasting humoral immune responses at the mucosal portal of virus entry. Hence, the design of a vaccine strategy able to induce mucosal antibodies and in particular specific IgA, may be crucial to providing immune protection. Nasal immunisation is known to induce specific IgG and IgA responses in the cervicovaginal mucosa; however, there is an urgent need for the development of safe, effective and accessible mucosal adjuvants for nasal application in humans. To reduce the potential for adverse events associated with some nasal adjuvants, we have assessed whether the B-cell-activating cytokines APRIL, BAFF and TSLP enhance humoral immune responses to HIV-1 gp140. Following intranasal immunisation, TSLP but not APRIL or BAFF induced strong humoral responses both in serum and mucosa. The adjuvant effect of TSLP on humoral responses was similar to that of cholera toxin (CT). The use of TSLP as an adjuvant skewed both the cellular and humoral immune responses towards Th2 cells. This is the first time that TSLP has been demonstrated to have a positive effect as a mucosal adjuvant, and specifically to promote mucosal and systemic responses to HIV gp140.

## Introduction

The predominant route of sexual HIV transmission to women is via the mucosa of the genitourinary tract. For this reason, there is a growing consensus that a vaccine is needed that can induce both cellular and humoral immunity in systemic and mucosal compartments 1. While research has focused on the induction of systemic CTL responses and neutralising antibodies, the induction of IgA at mucosal surfaces by vaccination has received less attention. This may provide alternative or additional protective function to the classical antibody neutralisation. In particular, mucosal IgA is: predominantly dimeric; more resistant to protease degradation than IgG isotypes; can aggregate and/or trap virus in cervical mucus, limiting direct contact with the underlying epithelium; is specifically secreted at mucosal surfaces through interaction with the poly-immunoglobulin receptor (pIgR) on columnar epithelial cells, can prevent epithelial transcytosis; and, where neutralising antibodies are induced, can facilitate intraepithelial cell neutralisation 2. As there are currently major challenges in the induction of broadly neutralising antibodies to HIV-1, it is thought that such immune exclusion functions of specific IgA could be exploited in a mucosal vaccine to augment conventional IgG neutralisation.

Nasal immunisation has long been reported as one of the most effective routes to induce immune responses in the female genital tract 3. It is easily accessible, may be more culturally accepted than the vaginal or rectal routes of immunisation, requires lower amounts of antigen to induce immune response, and has the ability to induce potent responses both in serum and mucosally 4. However, the magnitude of the immune response depends, to some extent, on immunogen, adjuvant, and delivery method 5. Currently, there is an important need for the development of safe and effective adjuvants for nasal application in humans. Due to inherent safety issues, cholera toxin (CT), the model mucosal adjuvant used for many animal studies, is not appropriate for human use, and detoxified versions of CT and the closely related heat-labile enterotoxins (LTs) from *Escherichia coli* also have safety concerns for intranasal use in humans 6. While studies have shown that a range of other adjuvants can promote intranasal immunisation with HIV envelope proteins (gp120, gp140 and gp160) in animal models 7–13, many of these adjuvants trigger multiple signalling pathways, which may not be central to their adjuvant effects, increasing the potential for unwanted side effects in humans. Furthermore, not only are mucosal responses per se often short-lived, antibody responses to HIV envelope proteins can rapidly wane in the systemic compartment after each immunisation 14–16. This only serves to highlight the pressing need to develop novel mucosal adjuvant strategies for HIV-1 envelope based vaccines.

A possible alternative approach to the induction of potent and enduring mucosal responses to HIV envelope proteins is the use of specific B-cell-associated cytokines such as thymic stromal lymphopoietin (TSLP), a proliferation-inducing ligand (APRIL) and B-cell-activating factor (BAFF), which are strong inducers of humoral responses 17. These may be potentially safer as they could directly target B cells and/or DCs without activating other redundant pathways, unlike the more pleiotropic effects of other adjuvants. TSLP is an IL-7-like 4-helix bundle cytokine of 140-amino acids that was originally shown to support B-cell development 18, 19. The induction of TSLP in mice is associated with several known TLR ligands (e.g. Poly I:C) and proinflammatory cytokines (e.g. IL-1α/β and TNF-α) 20. TSLP activates DCs, but also provides DCs with the ability to create a permissive environment for T_H_2 cell differentiation 21, which may promote the generation of antigen-specific IgA-producing B cells. This may be mediated in part through the induction of BAFF and APRIL augmenting class switching by intraepithelial B cells 20, 22. BAFF and APRIL are members of the TNF ligand superfamily. BAFF, and possibly APRIL, have been shown to be crucial factors involved in class switch recombination from Cμ to Cγ and/or Cα, with subsequent increase of IgG- and IgA-secreting cells, respectively 23. However, the use of such factors as adjuvants is not clear-cut. TSLP has been associated with allergy, particularly relating to the induction of IgE 24, while the induction of BAFF by gp120 binding to C-type lectin receptors has been proposed as a mechanism for polyclonal immunoglobulin class switching through a CD4^+^ T-cell-independent mechanisms 25.

In this study, we have investigated whether TSLP, APRIL and BAFF can be used as effective intranasal adjuvants for HIV-1 gp140. TSLP but not APRIL or BAFF induced strong and sustained serum and mucosal immune responses after nasal immunisation, comparable to those seen with CT. Intranasal, but not intradermal immunisation induced vaginal IgA responses, while both routes induced systemic IgG. Of note TSLP shifted the immune response towards a Th2-type response. These results suggest that TSLP may be a promising new intranasal adjuvant to enhance immune responses to gp140 and other nasal vaccines.

## Results

### TSLP induces specific immune responses after intranasal immunisation

**Figure 1:**
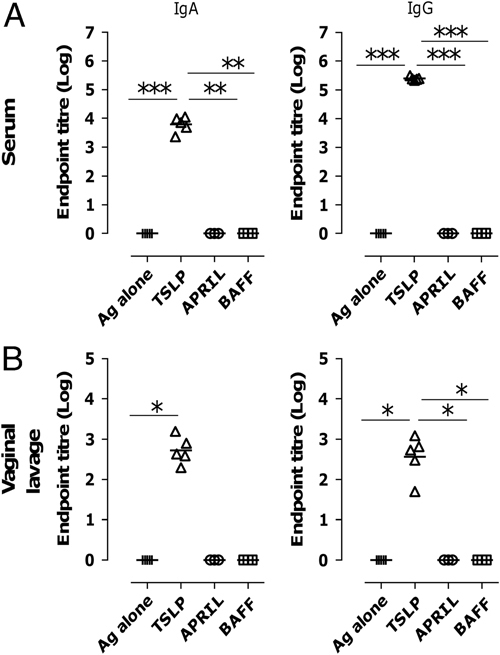
TSLP induces gp140-specific immune responses after intranasal immunisation. Mice were immunised with 10 μg gp140 in the presence or absence of 5 μg TSLP, APRIL or BAFF i.n. three times at 3 week intervals. Anti-gp140 IgA (left) and IgG (right) were measured 3 weeks after the final immunisation by ELISA in (A) serum and (B) vaginal lavage. Data are shown as endpoint titres, with lines representing geometric means of *n*=5 mice; data are representative of two experiments. ^*^p<0.05, ^**^p<0.01, ^***^p<0.001, one-way analysis of variance (ANOVA) with Tukey's multiple comparison post test.

We initially explored the potential of TSLP, APRIL, BAFF as mucosal vaccine adjuvants. Mice were immunised i.n. or intradermally (i.d.) three times at 3 week intervals in a prime-boost-boost protocol with 10 μg CN54gp140 alone or in the presence of 5 μg TSLP, APRIL or BAFF. Anti-gp140-reactive IgG and IgA were measured in serum and vaginal lavage collected at the end of the experiment. Mice immunised i.n. with gp140 plus TSLP but not APRIL or BAFF induced high titres of gp140-specific IgA and IgG in serum and vaginal lavage (Fig. 1A–D). Neither TSLP, APRIL nor BAFF had any adjuvant effect compared to gp140 alone when used i.d. (data not shown). Bioactivity of the APRIL and BAFF used was confirmed using a B-cell proliferation assay (data not shown).

The adjuvant effect of i.n. TSLP was dose dependent with maximal responses seen at 5 μg (data not shown). TSLP induced significantly higher systemic and vaginal responses to intranasal immunisation with ovalbumin (OVA) compared with OVA alone (data not shown) demonstrating that the adjuvant potential of TSLP was not antigen specific.

No side effects were observed during or after the administration of any of the immunisations (data not shown). We wished to determine whether intranasal TSLP was inducing an acute inflammatory response. Mice were i.n. immunised with CN54 gp140 alone or with CT or TSLP, i.n. LPS and PBS were used as controls. Six hours later mice were culled and cells from the bronchoalveolar lavage (BAL) recovered. There was no significant difference in cell recovery between groups (data not shown). While i.n. LPS exposure induced a significant recruitment of neutrophils, there was no difference between gp140:TSLP, gp140:CT or gp140 alone compared with the PBS controls (data not shown), with the BAL predominantly composed of macrophages. There were no observable differences in lung or nasal sections stained with either H&E or Periodic acid-Schiff (PAS).

### The adjuvant effect of TSLP on the humoral but not cellular response to gp140 is comparable to that of CT

**Figure 2:**
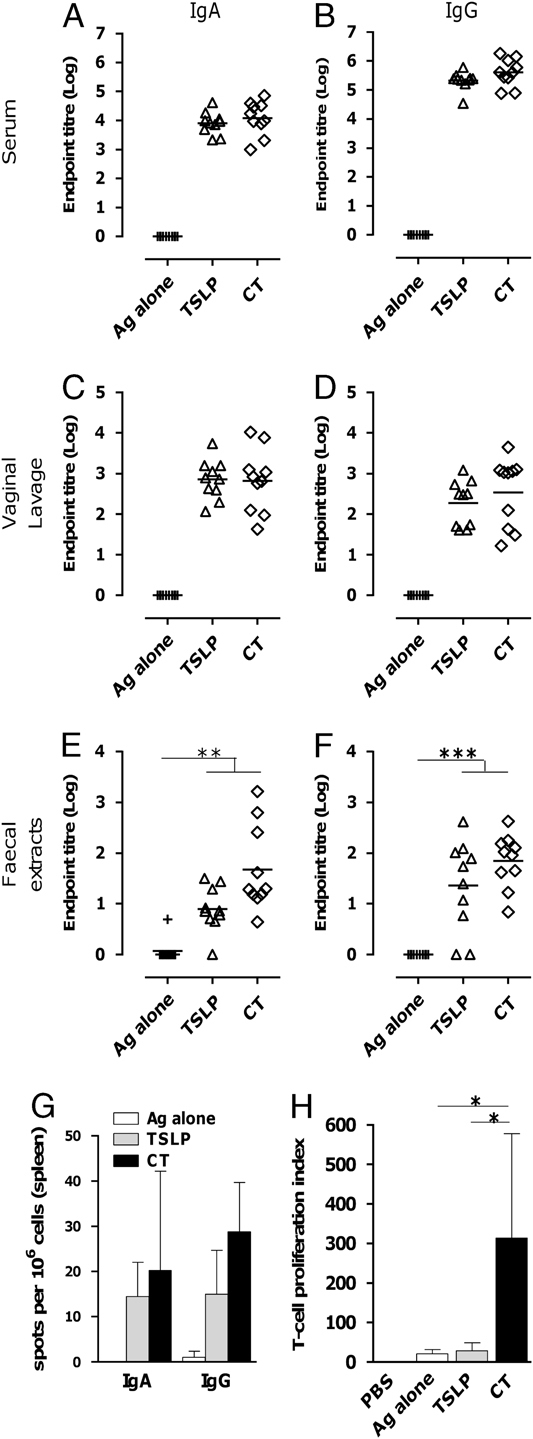
TSLP induces similar humoral but not cellular responses to CT. Mice were immunised three times i.n. with 10 μg gp140 alone (+),with 5 μg TSLP (△) or CT (◊). Anti-gp140 IgA and IgG were measured by ELISA in (A, B) serum, (C, D) vaginal lavage and (E, F) faecal extracts obtained from mice 3 weeks after the final immunisation. (G) Anti-gp140 IgA and IgG ASC were measured by B-cell ELISPOT in spleen from mice 3 wks after the final immunisation. (H) 10 days after the final immunisation, splenocytes were isolated and cultured for 5 days with CN54 gp140 protein and proliferation measured by ^3^H thymidine incorporation. Data points represent single mice, bars represent mean of *n*=5 mice+SD (G, H). Data are representative of two experiments. ^*^p<0.05, ^**^p<0.01, ^***^p<0.001, one-way analysis of variance (ANOVA) with Tukey's multiple comparison post test.

Having demonstrated that TSLP was the most effective cytokine adjuvant of those tested, we sought to compare it with CT a mucosal adjuvant commonly used in murine studies 3, 5. Mice were immunised three times i.n. with 10 μg gp140 alone or with 5 μg TSLP or CT. The strong humoral immune responses to gp140 induced by TSLP in serum and mucosally, vaginal lavage and faeces, was comparable to that observed with CT (Fig. 2). CN54 gp140-specific IgA and IgG antibody-secreting cells (ASC) were detected by ELISPOT in the spleen of animals immunised with gp140 plus TSLP or CT (Fig. 2G). Splenocytes were isolated and cultured with CN54 gp140 protein or a selected CN54 gp140 peptide pool. T-cell proliferation was measured by ^3^H thymidine incorporation. CT induced significantly more T-cell proliferation to both the protein (Fig. 2H) than antigen alone or TSLP. Both antigen alone and TSLP induced some cell proliferation.

### Immunisation with TSLP induces a Th2-skewed immune response

**Figure 3:**
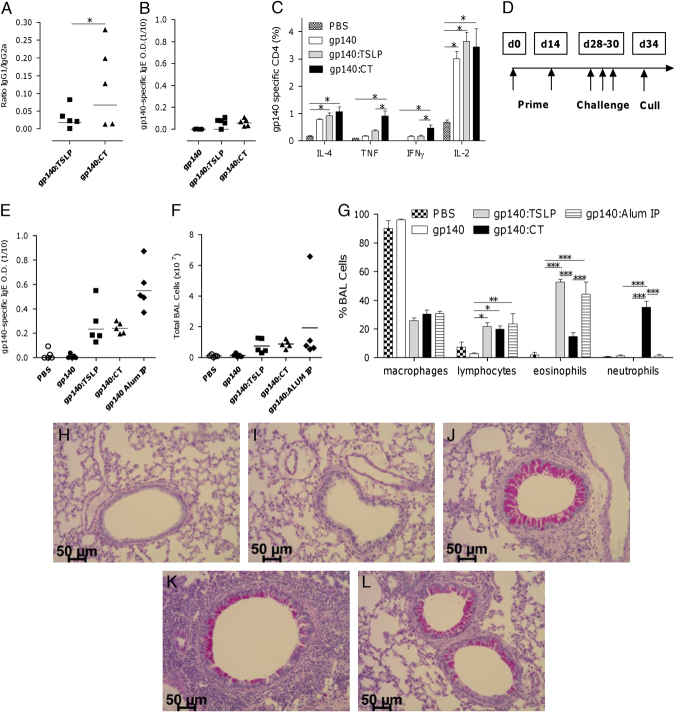
TSLP shifts the immune response to gp140 to Th2 Mice were immunised three times i.n. with 10 μg gp140 alone or with 5 μg TSLP or CT. (A) Anti-gp140 IgG_1_ and IgG_2a_ and (B) IgE were measured in serum by ELISA. (C) Splenocytes were stimulated with a CN54 gp140 peptide pool and specific cytokine production was measured in CD4^+^ T cells by flow cytometry. (D) Mice were immunised twice at 2 week intervals, followed by three daily i.n. challenges with 20 μg gp140 in 100 μL and were culled 3 days later. (E) gp140-specific IgE was measured in sera. (F) Cells were collected from bronchoalveolar lavage (BAL) and counted and (G) differential cell counts performed. Representative PAS-stained sections of lungs from (H) PBS, (I) gp140, (J) gp140:TSLP, (K) gp140:CT, (L) IP gp140:alum. Data are shown as mean of *n*=5 mice+SD, experiment performed once. ^*^p<0.05, ^**^p<0.01, ^***^p<0.001, one-way analysis of variance (ANOVA) with Tukey's multiple comparison post test.

TSLP has been associated with a shift in the immune response towards Th2-type responses 26. Mice were immunised three times i.n. with 10 μg gp140 alone or with 5 μg TSLP or CT. The use of TSLP as an adjuvant induced a shift in the gp140-specific IgG subtype response towards IgG1, which is associated with Th2-cell responses, compared to CT (Fig. 3A). Both TSLP and CT led to a small, but not statistically significant, level of gp140-specific IgE detectable following three immunisation (Fig. 3B). We measured intra-cellular cytokine expression to a CN54 peptide pool in both CD4^+^ and CD8^+^ T cells (sample plots in Supporting Information Fig. 1). Antigen alone, TSLP plus antigen and CT plus antigen all induced significant levels of gp140-specific IL-2 production in both CD4^+^ and CD8^+^ T cells and IL-4 in CD4 cells. CT plus antigen also induced significant levels of TNF, IFN-γ and IL-17 expression by CD4^+^ T cells (Fig. 3C).

To further characterise the Th2-cell skewing of immune responses following immunisation with TSLP plus gp140, we adapted a model of allergic airway disease. It has been previously shown that immunisation of mice with antigen (normally ovalbumin) and alum i.p. can induce a strongly pro-Th2-cell response, with IgE, airway eosinophilia and goblet cell hyperplasia. We therefore used i.p. gp140 and alum as a positive control compared to i.n. immunisation with PBS, CN54 gp140 alone or with CT or TSLP. Mice were i.n. immunised (using a 20 μL volume) twice, at 2 week intervals, followed by an exacerbated airway challenge of three i.n. doses of 20 μg gp140 alone (in a 100-μL volume to maximise airway exposure) and subsequently culled 3 days later (Fig. 3D).

Following challenge, mice immunised with gp140 and an adjuvant had significantly higher production of gp140-specific IgE than mice immunised with gp140 alone. Levels of IgE were significantly higher in mice immunised with gp140:alum than gp140:TSLP or gp140:CT (Fig. 3E). Mice immunised with gp140 plus adjuvant had a greater total cell recruitment to the airway than mice immunised with PBS or gp140 alone (Fig. 3F). The BAL of mice immunised with PBS or gp140 alone was predominantly comprised macrophages (Fig. 3G). There were significantly more lymphocytes recruited to the airways of mice immunised with adjuvant (regardless of adjuvant). Mice immunised with gp140:CT had a significant neutrophilia compared to all other groups. Mice immunised with gp140:TSLP or gp140:alum had significantly more eosinophilia than the other groups. Staining of lung sections with PAS showed that immunisation with PBS (Fig. 3H) or gp140 (Fig. 3I) alone did not lead to the development of goblet cell metaplasia around the major airways. However, the use of an adjuvant led to a goblet cells metaplasia and mucous production (pink-stained cells). Similar PAS-stained cells were detected in the gp140:TSLP (Fig. 3J) and gp140:alum (Fig. 3L) groups, with slightly less staining in the gp140:CT (Fig. 3K) group. Immunisation with gp140:CT led to a greater infiltration around blood vessels and large proximal airways than in other groups, using H&E staining it was seen that this infiltrate had a larger proportion of granulocytes than other groups. From all of these data, it can be concluded that TSLP does skew the immune response towards a Th2-cell-type response.

### I.n. immunisation induces higher gp140-specific IgA responses, but not IgG, than intradermal immunisation

**Figure 4:**
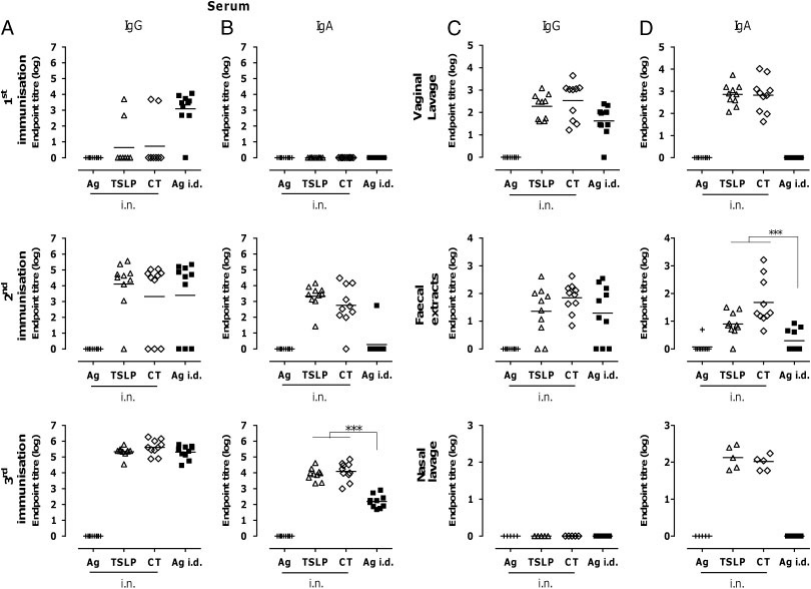
I.n. immunisation induces higher gp140-specific IgA responses than i.d immunisation. Mice were immunised i.n. (open symbols) or i.d. (black symbols) three times with 10 μg gp140 alone or with 5 μg TSLP or CT. (A, C) Anti-gp140 IgG and (B, D) anti-gp140 IgA were measured by ELISA (A, B) in serum 3 weeks after priming (first immunisation), first boost (second immunisation) and final immunisation (third immunisation) and (C, D) in vaginal lavage, faecal extracts and nasal lavage 3 weeks after the final immunisation. Data are shown as endpoint titres, with lines representing geometric means of *n*=5 mice; data are representative of two experiments. ^***^p<0.001, one-way analysis of variance (ANOVA) with Tukey's multiple comparison post test.

To compare the effect of route of immunisation on the kinetics and distribution of the antibody responses, mice were immunised i.n. or i.d. with gp140 and adjuvants. Intradermal immunisation induced gp140-specific serum IgG responses earlier than i.n. immunisation with significant seroconversion after the first immunisation (Fig. 4A). Serum anti-gp140 IgG titres reached similar levels after the second immunisation regardless of the route (Fig. 4A). In contrast to IgG, i.n. immunisation induced gp140-specific serum IgA responses more rapidly than i.d. immunisation and led to significantly higher values (*p*<0.001, Fig. 4B). In vaginal lavage and faecal extracts, similar levels of specific gp140 IgG were observed after immunisation by either route (Fig. 4C). However, only i.n. immunisation induced high titres of specific gp140 IgA in mucosal samples (Fig. 4D). No IgG was detectable in nasal lavage, but gp140-specific IgA was detected after i.n. immunisation.

### TSLP induces durable humoral and cellular responses to intranasal immunisation with gp140

**Figure 5:**
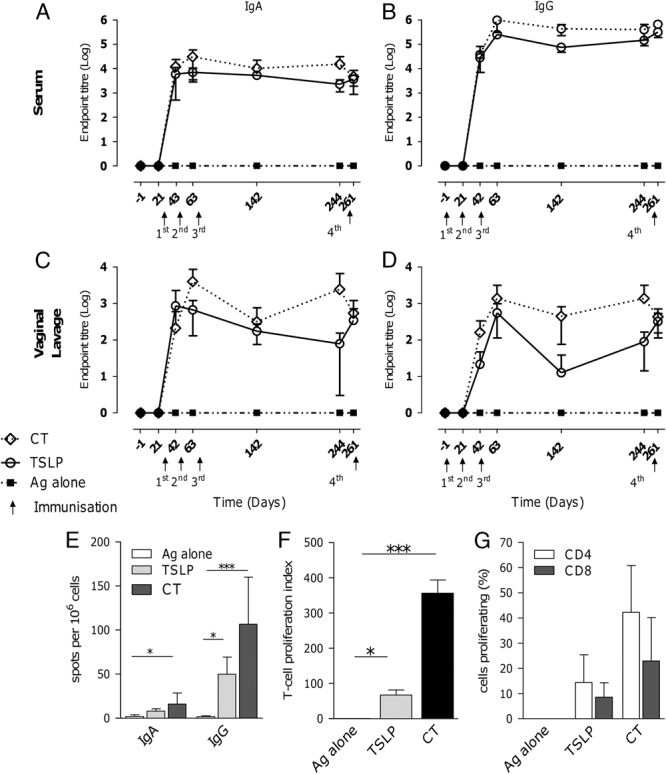
TSLP induces sustained humoral and cellular immune responses to gp140. Mice were immunised four times i.n. with 10 μg gp140 alone (▪) or with 5 μg TSLP (○) or CT (◊). The fourth immunisation was given 6 months after the third immunisation. Anti-gp140 IgA and IgG were measured by ELISA in (A, B) serum and (C, D) vaginal lavage before every immunisation and 3 weeks after the final immunisation. (E) CN54 gp140 IgA and IgG ASC were measured by B-cell ELISPOT in spleens from mice 3 weeks after the final immunisation. Antigen-specific T-cell proliferation of splenocytes was assessed after in vitro stimulation with gp140 after 120 h using (F) ^3^H-thymidine and (G) CFSE dilution. Data are shown as mean of *n*=5 mice+SD, experiment was performed once. ^*^p<0.05, ^**^p<0.01, ^***^p<0.001, one-way analysis of variance (ANOVA) with Tukey's multiple comparison post test.

To investigate whether the responses to gp140 induced by TSLP were durable, mice were immunised i.n. three times at 3 weeks intervals and then given a fourth immunisation 6 months later. Serum specific IgA and IgG levels were sustained for 6 months after the third immunisation and there was minimal impact of a fourth immunisation (Fig. 5A and B). Specific IgA and IgG levels in vaginal lavage of animals immunised i.n. with gp140 plus TSLP appeared to decrease slightly in the course of 6 months, but this trend was only significant for IgG with TSLP (*p*<0.05). Boosting at 6 months restored these responses to levels seen after the third boost at day 63 (Fig. 5C and D).

Spleens were collected after the final immunisation, and gp140-specific IgA and IgG B-cell ELISPOT assays were performed. gp140-specific IgA- and IgG-ASC were induced in the spleen after i.n. immunisation (Fig. 5E). TSLP and CT significantly induced splenocyte T-cell proliferation responses to gp140 (Fig. 5G), but CT responses were greater than TSLP. Both adjuvants induced CD4^+^ and CD8^+^ T-cell proliferation measured by CFSE (Fig. 5H).

## Discussion

This study assessed the capacity of the B-cell-activating cytokines APRIL, BAFF and TSLP to enhance humoral and cellular immune responses to HIV-gp140 after intranasal immunisation. TSLP, but not APRIL or BAFF, was able to induce strong, long-lasting antibody responses both in serum and mucosally, including the vaginal compartment. These responses were comparable to those induced by the established experimental mucosal adjuvant CT. In addition, intranasal immunisation induced higher gp140-specific IgA responses than intradermal immunisation without compromising levels of specific IgG levels in serum. IgA and IgG producing gp140-specific B cells were detected in the spleen. TSLP also induced T-cell responses to gp140, with specific CD4^+^ T-cell responses greater than CD8^+^ T-cell responses.

Although the previous studies have shown that systemic responses to HIV envelope protein (Env) rapidly wane in humans after each immunisation 14–16, TSLP induced sustained systemic IgG and IgA responses in mice that showed no diminution over a six-month period. Furthermore, while mucosal responses appeared to show a trend towards a modest reduction over this period, these were restored following a booster immunisation at six months. It is unclear whether these results will directly translate to humans, nevertheless the observed duration of humoral response is encouraging. As the main focus of this study was to determine the impact of routes of administration on specific humoral responses, we did not assess neutralisation responses. This has however been previously characterised for the CN54 gp140 in rabbits 27, thought to be a more appropriate model than mice for assessment of neutralisation breadth.

The specific mechanisms underlying the adjuvant affect of TSLP on the immune response to gp140 have not been defined, but based on its known properties we speculate that TSLP may directly activate DCs situated within the nasal mucosa to take up antigen via dendrites that protrude the nasal epithelium 28. TSLP licensed DCs could then activate T-helper cells inducing B-cell activation and differentiation 21, 29–31 or act directly on B cells by producing BAFF and/or APRIL 22, 23, 32. TSLP has been shown to boost DC priming of CD8^+^ T cells following BCG exposure in vitro 33. Epithelial cells 34, basophils 35 and M cells may also potentiate the adjuvant action of TSLP.

In addition, TSLP, in combination with TCR stimulation, can act directly on naïve CD4^+^ T cells to promote Th2-cell differentiation 36, 37, proliferation and survival of effecter Th2 cells 38. Supporting this mechanism, we observed a Th2 bias in the cellular and Ig response to gp140 when delivered with TSLP. The Th2 skewing of antigen-specific responses induced by TSLP may be a possible safety concern for further development of TSLP as an adjuvant. The observation that TSLP could induce eosinophilia in a model of allergy airway disease is consistent with previous studies demonstrating that i.n. immunisation every other day for 2 weeks using 0.5 μg TSLP plus OVA induced allergic airway disease 39. It is unclear as to the physiological relevance of such chronic exposure to discrete immunisations. Indeed, using a conventional prime, boost, boost immunisation strategy there was no significant antigen-specific IgE production compared to antigen alone or CT and the absolute amount of specific IgE was considerably less than specific IgG or IgA. Furthermore, a single immunisation with TSLP and antigen failed to induce goblet cell metaplasia or acute infiltration in the airways.

The induction of antigen-specific T cells by TSLP is a critical step in the induction of a strong antibody response and may account for the difference in the adjuvant activity observed between TSLP and BAFF/APRIL. Thus, whereas TSLP acts upon DCs and T cells to induce a stronger Th2 phenotype 21, 37, BAFF and APRIL act directly on B cells promoting T-cell-independent responses 40. The observed lack of response to APRIL alone contrasts with a previous study suggesting that APRIL enhanced i.n. immunisation with OVA in BALB/c mice 7, however this former study utilised human APRIL in the mouse model, shown to bind murine receptors BCMA and TACI 41, and therefore direct comparisons cannot be made.

Importantly, the adjuvant effect of TSLP described here does not appear to be antigen specific as it was also observed to enhance immune response to i.n. immunisation with OVA (data not shown). Such data suggest the adjuvant properties of TSLP may be broadly applicable. We also evaluated the ability of TSLP to induce specific responses by additional mucosal routes. Interestingly, whereas sublingual immunisation-induced responses were almost as potent as intranasal immunisation (data not shown), intravaginal immunisation using the same dose of gp140 plus TSLP failed to induce any detectable response (mucosal or systemic). However, we have shown that immunisation via the vaginal route in mouse requires a systemic prime 42.

Here, we show for the first time that TSLP has the capacity to act as a mucosal adjuvant-inducing antibody and cellular responses to gp140. This suggests that the role of TSLP in promoting aberrant and protective immune responses is likely to be both context and antigen specific 26. Thus, the potential role of TSLP in induction of protective mucosal responses to mucosal pathogens, and to gp140 in particular, may offer important insight to the induction of natural and/or vaccine mediated mucosal immunity.

## Materials and methods

### Adjuvants

Carrier-free recombinant mouse TSLP and BAFF were purchased from R&D systems (USA) and APRIL was purchased from PeproTech (USA). CT from *Vibrio cholerae* was purchased from Sigma-Aldrich (UK). Cytokines had no detectable levels of endotoxin, testing of samples by Lonza, Basel, Switzerland. Bioactivity of BAFF and APRIL was tested on primary murine splenocytes.

### Antigen

Trimeric recombinant gp140 (gp120 plus the external domain of gp140) envelope protein from the HIV-1 clade C strain CN54 was produced as a recombinant product in CHO cells and manufactured to GMP specification by Polymun Scientific, Vienna, Austria. The clade C HIV-1 envelope clone p97CN54 was originally isolated from a Chinese patient 43 and was made available by H. Wolf and R. Wagner, University of Regensburg, Germany.

### Vaccine preparation and immunisation of mice

Female BALB/c mice were obtained from Harlan Olac (Epsom, UK) and used when 6- to 10-weeks old. All work was performed according to the Institutional and Home Office guidelines. Ten micrograms HIV-1 gp140 were mixed with PBS alone or in the presence of 5 μg TSLP, APRIL, BAFF or CT.

The final volume in PBS for i.n. and i.d. administration was 20 and 50 μL, respectively. For i.n. immunisation, mice were sedated using isoflurane and 10 μL of vaccination preparation was slowly applied to each nostril using a micropipette.

For the airway challenge model, mice were immunised twice at 2-week intervals, followed by three i.n. challenges at daily intervals with 20 μg gp140 in 100 μL and were culled 3 days later (Fig. 3D).

### Sample collection and processing

Samples were obtained at pre-immunisation and 21 days after each immunisation.

*Blood/sera*. Blood was collected by tail bleeding and at the end of the experiment by cardiac puncture. Sera were collected from centrifuged, coagulated blood.

*Vaginal samples.* Samples were collected by gentle washing of the vaginal mucosa with 25 μL of PBS. This procedure was repeated three times after which the washes were pooled and placed on ice for 30 min in the presence of 5 μl of 25× protease inhibitor cocktail (Roche Diagnostics, Germany). After incubation, the samples were spun at 13 000×*g* for 10 min, the supernatants were collected and stored at −80°C until further use.

*Faecal samples.* Ten faecal pellets were mixed with 400 μL PBS+1× protease inhibitor cocktail per 100 mg faeces, homogenised by vortexing, and incubated for 1 h on ice. The samples were centrifuged twice at 13 000×*g* for 10 min, the supernatants were collected and stored at −80°C.

*Nasal lavage.* Nasal samples were obtained at the end of the experiment by flushing the nasal passage with 300 μL of PBS+1× protease inhibitor cocktail and stored at −80°C.

*BAL*. BAL samples were collected and processed as described previously 44. Cells were enumerated by haemocytometer and differential cell counts performed using H&E-stained cytospins.

*Lungs for fixation*. Histology samples were processed from 4%-formalin-fixed lungs and stained with hematoxylin and eosin (H&E), and mucus-producing goblet cells were detected using PAS.

*Splenocytes*. Splenocytes were isolated by mechanical dissociation through sterile nylon mesh, followed by red blood cell lysis. Cells were always kept on ice unless specified otherwise.

### Detection of antigen-specific antibody responses by ELISA

MaxiSorp 96-well plates were coated overnight with 5.0 μg/mL HIV-1 gp140 in PBS. Plates were blocked for 1 h at 37°C with 1% BSA in PBS. Serially diluted samples were incubated for 1 h at 37°C. Bound IgA and IgG was detected by incubation for 1 h at 37°C with horseradish peroxidase (HRP)-conjugated goat anti-mouse IgG, IgE, IgG_1_, IgG_2a_ (AbD Serotec, UK) or biotin-conjugated goat anti-mouse IgA and IgE (AbD Serotec, UK). For IgA and IgE detection, plates were incubated with Streptavidin-HRP (R&D Systems) for 1 h at 37°C. Plates were developed using tetramethylbenzidine (TMB) substrate. The reaction was stopped with stop solution (1 N H_2_SO_4_) and read at 450 nm. Reciprocal endpoint titres were calculated by using the GraphPad Prism 4 using a cut-off value at OD_450_ of 0.1 for IgG and serum and vaginal IgA, and 0.5 for faecal IgA.

### Ex vivo T-cell proliferation assays

Splenocytes were cultured in the presence of 5 μg/mL gp140 for 120 h. Concanavalin A (Con-A from *Canavalia ensiformis*, Sigma-Aldrich) was used at 5 μg/mL as a positive control of stimulation, and medium alone as a negative control. The T-cell proliferation response was measured by [^3^H] thymidine incorporation into the DNA or CFSE staining.

*^3^H-thymidine.* 0.5 μCi [^3^H] thymidine (GE Healthcare, UK) was added per well 16–18 h before the end of the experiment. Plates were harvested using a Harvester 96 (TOMTEC) and the counts per min (c.p.m.) were counted in a 1450 Microbeta Plus Counter (Wallac Oy, Finland). The T-cell proliferation response was expressed as the stimulation index, which was calculated by dividing the experimental c.p.m. (HIV-1 gp140) with the background c.p.m. (medium). A stimulation index ≥3 was considered as a positive proliferation response.

*CFSE.* One million cells were stained with 2 μM CFSE at time zero, according to manufacturers' recommendations (CellTrace™ CFSE Cell Proliferation Kit, Invitrogen). After incubation with antigen, the cells were stained with APC-H7-anti-CD8, Allophycocyanin-anti-CD4 and PE-Cy5-anti-CD3 (BD) in addition to staining with CFSE. Cell viability was assessed with the LIVE/DEAD® Fixable Violet Dead Cell Stain Kit (Invitrogen). Data acquisition was performed using a FACS CANTO II cytometer (BD Biosciences, San Jose, CA, USA), and the data analysed using the FACS DIVA software version 6.1.3 (BD Biosciences).

### Intracellular cytokine staining

Cells were stimulated for 6 h with a peptide pool consisting of 39 peptides and 2 μg/mL CD28 (BD Pharmigen), 50 ng/mL PMA and 500 ng ionomycin (Sigma) or medium alone. Peptides were selected from a bigger pool of 156 peptides covering the whole of HIV-1 CN54 gp140 for their ability to restimulate splenocytes in vitro after immunisation. Protein transport inhibitor (containing Monensin, PB GolgiStop) was added at the beginning of stimulation for peptides and medium alone and 2 h later for PMA and ionomycin stimulation. Cells where labelled for surface antigens using Pacific Blue anti-mouse CD3e (BD Pharmingen), V500 anti-mouse CD4 (BD Horizon), PerCP anti-mouse CD8a (BioLegend) and blocked using purified rat anti-mouse CD16/CD32 (Fc Block, BD Pharmingen). After staining, cells where washed, fixed and permeabilised using BD Fixation/Permeabilisation Kit. Intracellular cytokine staining was accomplished using PE-Cy7 anti-mouse IL-4, PE anti-mouse IL-17A, allophycocyanin-Cy7 anti-mouse IL-2 (all BD Pharmingen), FITC anti-mouse TNF-α and allophycocyanin anti-mouse IFN-γ (BioLegend). Appropriate isotype controls were used in all steps. Staining specificity was confirmed using fluorescence minus one (all Abs minus one). Analysis were performed onto a FACS CANTO II cytometer, using FACS DIVA software (BD Biosciences).

### B-cell-ELISPOT

HIV-1 gp140 IgA-or IgG-specific B-cell-ELISPOT using ELISpot PLUS for mouse IgA and IgG following manufacturers' recommendations (MABTECH, Sweden). For HIV-1 gp140-specific responses, plates were coated overnight with 20 μg gp140/mL in PBS. Splenocytes were pre-cultured for 3 days with 5 μg lectin from *Phytolacca americana* (pokeweed) (Sigma) and 10 ng recombinant mouse IL-2 (R&D systems) prior to overnight incubation on the coated ELISPOT plates prior to development.

### Statistical analysis

Analyses were performed using the GraphPad Prism, version 4.00 (GraphPad Software). Statistical differences between groups were calculated using one-way analysis of variance (ANOVA) with appropriate post tests to measure significance between pairs of groups.

## Supporting information

Detailed facts of importance to specialist readers are published as ”Supporting Information”. Such documents are peer-reviewed, but not copy-edited or typeset. They are made available as submitted by the authors.
