# Supplementary material for: Thymic stromal lymphopoietin (TSLP) acts as a potent mucosal adjuvant for HIV-1 gp140 vaccination in mice
Source: Eur J Immunol. 2011 Nov 7;42(2):353–63. doi: 10.1002/eji.201141787 (PMC3378695; doi:10.1002/eji.201141787)
Supplement: Supplementary file 1 [file eji0042-0353-SD1.pdf]

European Journal of Immunology

Supporting Information

for

DOI 10.1002/eji.201141787

Thymic stromal lymphopoietin (TSLP) acts as a potent mucosal adjuvant for HIV-1 gp140 vaccination in mice

Griet A. Van Roey, Mauricio A. Arias, John S. Tregoning, George Rowe and Robin J. Shattock

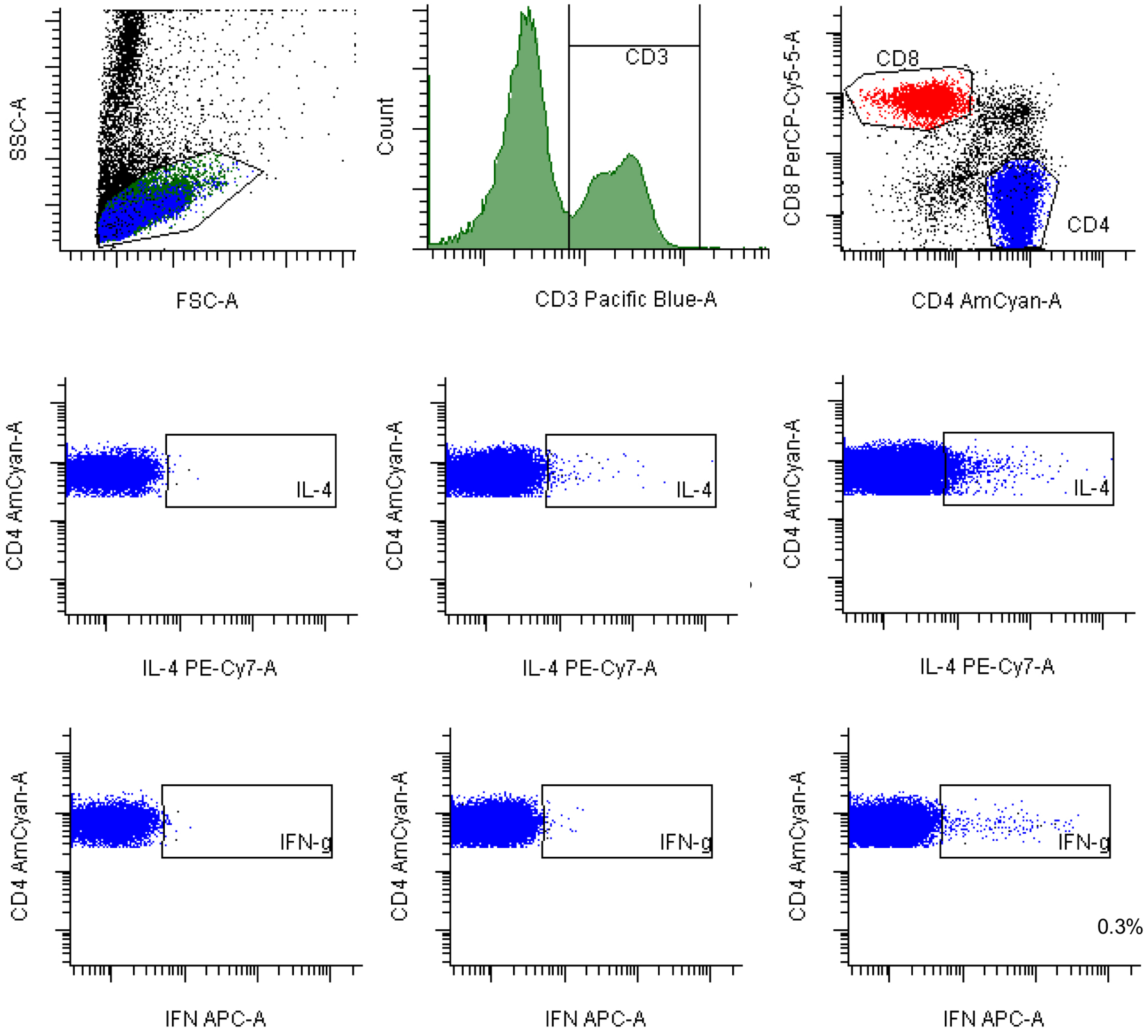

Supporting Information Figure 1. Sample plots from T cell intracellular cytokine stimulation. Figure shows flow cytometry data of (A) the gating strategy, (B) IL-4 staining and (C) IFN-γ staining from a representative mouse immunised 3 times i.n. with 10 µg gp140 5 µg CT. (B and C) For the intracellular cytokine staining, plots are shown of Fluorescence Minus One (FMO) which was used to set the gate, medium alone and gp140 peptide pool stimulation.
